# High-resolution global pathways to achieve 100% electricity access in 2030

**DOI:** 10.1038/s41598-025-23857-4

**Published:** 2025-11-17

**Authors:** Victhalia Zapata, Anteneh G. Dagnachew, Oreane Y. Edelenbosch, Detlef P. van Vuuren

**Affiliations:** 1 Copernicus Institute of Sustainable Development, Utrecht University, Utrecht, The Netherlands; 2 PBL Netherlands Environmental Assessment Agency, The Hague, The Netherlands

**Keywords:** Energy and society, Sustainability, Climate-change mitigation

## Abstract

Achieving universal access to a sufficient electricity supply is a crucial component of the Sustainable Development Goals. However, model projections suggest that under current policies, this goal will not be reached by 2030. There is still little understanding of the possible electrification strategies and associated costs across global regions. To address this gap, we explore scenarios for achieving universal electricity access globally in 2030 based on high-resolution data and energy projections from the integrated assessment model IMAGE. The scenarios consider baseline development, implementation of electricity supply per household consistent with decent living standards and synergies with climate change mitigation. The results indicate that off-grid systems, i.e. mini-grids and solar home systems, are the least-cost solution for most people gaining access after 2023 (base year). Furthermore, targeting universal access leads to at least 70GW of off-grid additional capacity needed, most of which is required for Sub-Saharan Africa. If combined with climate goals, the optimal strategy increases renewables, reducing CO_2_ emissions by around 30%, while system cost remains similar to the scenarios without climate policies.

**Supplementary Information:**

The online version contains supplementary material available at 10.1038/s41598-025-23857-4.

## Introduction

Globally, 675 million people still lack access to electricity. The majority of them live in Sub-Saharan Africa (SSA)^1^. Current projections indicate that under current trends in 2030, a similar number of people will still lack access to electricity^1^, meaning that the Sustainable Development Goal (SDG7.1) of universal electricity access will not be achieved. In fact, the progress in electrification has slowed down in recent years because of the increasing complexity of reaching more remote and poorer areas and the effect of the COVID-19 pandemic^1^.

Most research on universal access to electricity is focused on SSA^2–5^, given the dominance in the number of people lacking access in this region. However, this means that only a few studies have focused on the situation outside SSA, despite the fact that nearly 90 million people lack access^6–8^. Furthermore, a global perspective on electrification is necessary to understand the challenge and allocate resources accordingly.

Earlier studies have looked at the required costs for electrification, identifying both the required expansion of capacity and cost needs^4,9,10^. However, these studies suffer from a relatively low geographic resolution (sometimes regions) – which implies that heterogeneity in demand density and local renewable potential cannot be captured well. In the assessment of electricity access worldwide, it is important to cover these local variations^11^, especially for least-cost optimised models. The combination of high-resolution (HR) spatial assessment with integrated global modelling can provide insight into the possible future trade-offs and synergies between universal electricity access and climate change mitigation. Electricity access also needs to be seen in the context of essential needs. The World-Bank Multi-Tier Framework^12^ provides the option to define access from Tier 1 (sufficient for a few hours of lighting, phone charging and radio) to Tier 5 (capacity sufficient for air conditioner, refrigerator, ironing, washing machine, among others). Access scenarios often only cover Tier 1 demand level^1^. While achieving Tier 1 access can be a catapult to better lives, it is still insufficient to cover all needs for a decent living. In this research, we implement the definition of decent living given by Rao & Min^13^ with a focus on electricity demand for appliances needed in the household. The appliances are refrigerators, lighting, modern cooling if needed, one phone per household, one television or computer monitor, and one washing machine per household^13^.

This means that we add to the existing literature by 1) conducting an integrated analysis of least-cost strategies for achieving universal electricity access between 2024 and 2030 at the global scale, 2) considering decent living access levels, 3) identifying the required cost levels, and 4) looking into the synergies and trade-offs with climate change mitigation. We analyse differences across the model regions by considering spatially detailed parameters that can influence the electrification process (see methods). For this, we build upon the work of Dagnachew et al. for SSA^3^ and expand the geographic scope to the global level. Furthermore, the electricity access model is updated and re-coded for open-source access, and the spatial resolution is increased from 30’x30’ to 5’x 5’. This model is linked with the integrated assessment model IMAGE for implementing regional socio-economic data scenario projections and for assessing the climate impact of universal access strategies. The levelized cost (LCOE) for plausible electrification solutions is assessed per grid cell worldwide to select the least-cost option. The electrification solutions are grouped into central grid extension and two off-grid options: mini-grids and stand-alone systems. The stand-alone systems include solar home systems and diesel generators. The mini-grids considered are sourced by wind and PV (backed up by batteries), mini-hydropower, diesel, and PV and wind combined with diesel.

## Methods

### The household electrification model

**Fig. 1:**
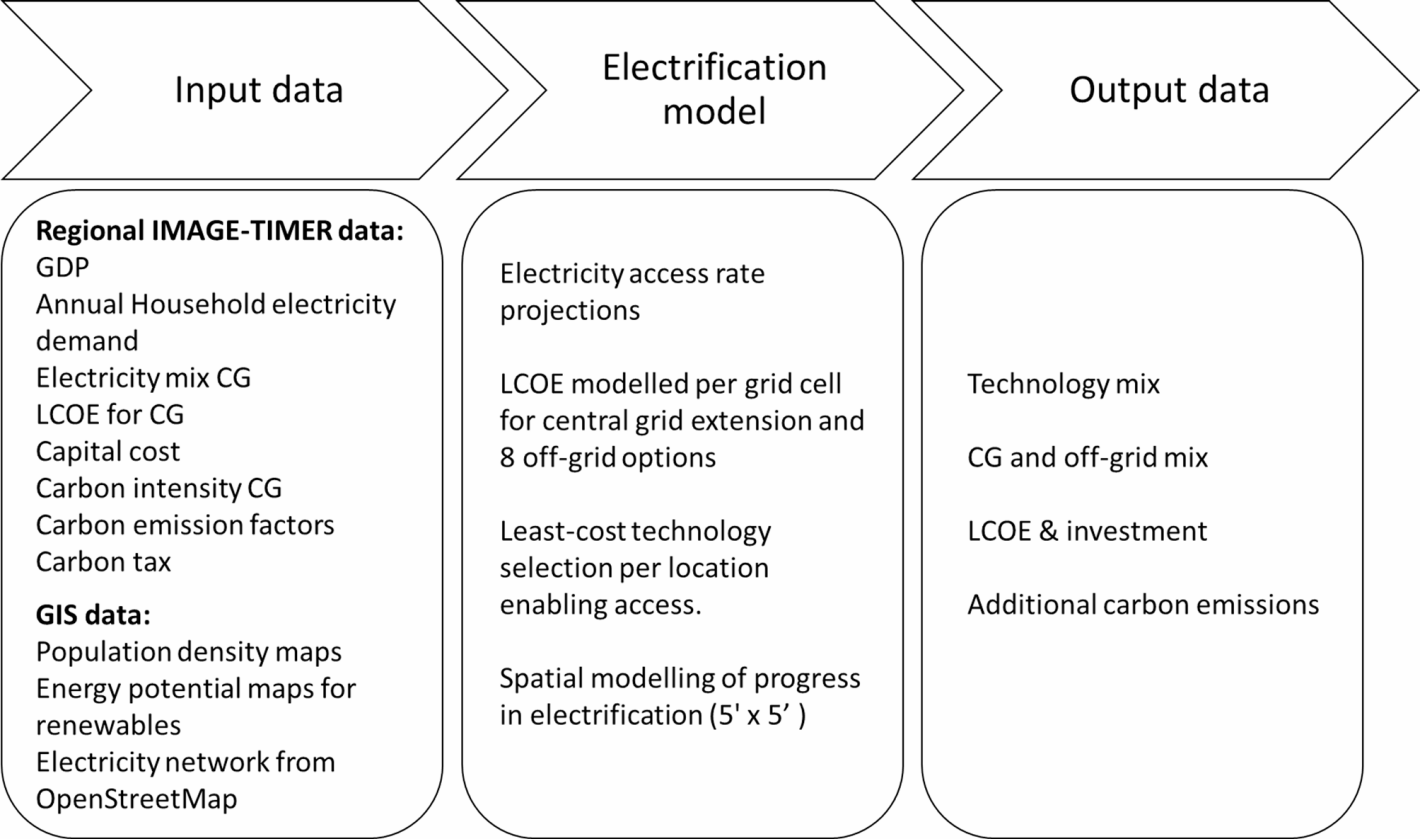
Schema of the methodology implemented (CG stands for central grid connections).

Figure 1 illustrates the general methodology implemented. The global spatial electrification model assesses the LCOE for grid extension (following Van Ruijven’s method^9^ or several off-grid options to select the least-cost option. The spatial resolution has been increased to 5’x5’ with a yearly temporal resolution until 2030. One least-cost solution is selected per grid cell; hence, increasing the resolution enables more locations to be sourced by renewables. In previous studies^3,14^, demand was aggregated at a larger area, making a certain local resource not enough to provide the demand or making the population-demand density too low, favouring stand-alone systems (photovoltaic home systems and diesel generators).

Annual electrification rates for each IMAGE region are first calculated using the van Ruijven method^9^, in which a multivariate function was implemented depending mainly on GDP, urbanisation rates, and population density. Then, the access rate is calibrated using the World Bank statistics until 2023^15^. These rates and a map with the distances to the electricity network are used to get the locations that already have access and to calculate the cost of providing access through a central network. We use a binary approach for access, which means that it is assumed that all people in a grid cell either have or do not have access. The distance to the electricity network is calculated using the available data by 2022 in OpenStreetMap^16^ of the electricity network at a resolution of 30”x30” and upscaled to the model resolution (5’x5’), weighting with population GIS data.

For the LCOE analysis, future electricity use per grid cell is estimated using spatial projections of urban and rural populations and regional data from the IMAGE model. This included average household size and electricity use for the rural and urban populations. The cost of grid extension is then calculated by estimating the required length of transmission and distribution lines per grid cell. This is determined based on maps of annual electricity use, projected peak demand, inhabited areas and household counts. Additionally, technical and cost data of the high-, medium-, and low-voltage lines and transformers are incorporated in the calculation^9^. The LCOE for off-grid technologies is calculated following the approach of Dagnachew et al.^3^. The required installed capacity is estimated based on the capacity factor for each technology. The capacity for wind and solar sources is calculated based on the average annual load, with batteries assumed to supply the extra peak load and during night hours. The LCOE per grid cell for hydropower and the capacity factor and potential maps for solar and wind were obtained from the work of Gernaat et al.^17^.

The least-cost electrification option, which requires deciding between central grid or off-grid options, is selected by an optimisation model. To make this choice, a distance threshold is calculated that indicates the maximum distance at which the levelised cost of grid extension is lower than the cheapest off-grid option. Furthermore, all locations within 50 km of the central grid are designated for grid extension to reduce the investment risk for off-grid investors^3^. The choice between stand-alone and mini-grid options is based on minimising costs. Additionally, for stand-alone systems, an upper limit on consumption density (in kWh/km2/year) is applied^3^. Within a region, it is assumed that locations with the lowest electrification cost get access first.

### IMAGE model and scenario development

IMAGE is an integrated assessment model representing different aspects of the global environmental and human system, including climate, land use, energy, and economics. It is designed to explore a wide range of future scenarios based on different assumptions about population growth, economic development, energy use, and climate policies. It divides the World into 26 regions (Supplementary Information, Figure S1) and has an annual temporal resolution. The regions relevant to this assessment are located in Sub-Saharan Africa, Central America, South America, Asia and Oceania^18^. This research focuses on its energy module, TIMER. The regional data used in the electrification model are CO_2_ emissions from the power sector, electricity produced per energy source, electricity demand per household and household size. The market share of electricity-producing technologies is selected using a multi-logit function, prioritising the cheapest technologies considering storage requirements for solar and wind sources^19^. Electricity demand for the residential sector is calculated through a bottom-up model, with detailed end-use representation (including light, cooling, heating and appliances) and by dividing the rural and urban populations into five income quantile groups. The demand is driven by population size, floor space, appliance ownership, efficiency, and climate conditions for cooling, heating and refrigeration^20^.

For analysing electrification pathways, the scenarios explored combine the shared socio-economic pathways 2 (SSP2) with and without climate mitigation strategies, two household electricity demand levels, and two future trends in access rate (see Table 1). The SSP2 is the middle-of-the-road pathway with median assumptions on economic, population growth and technological progress^21^. The baseline electrification rate depends on projected regional socio-economic conditions as described in van Ruijven et al.^9^. The universal access (UA) scenario follows a linear trend for achieving the goal by 2030, with the condition that the access rates for this scenario are equal to or larger than the baseline annual rates.

The household electricity demand levels explored are the baseline SSP2 levels and a scenario for targeting minimum decent living standard (DLS) demand. The standards are based on the work of Rao & Min, who define a broad set of material needs for having a decent living^13^. Here, we focus on those material needs driving electricity demand within the household. The scenario ensures that everyone has access to food refrigeration, lighting, modern cooling, one phone per household and one television/computer monitor per household^13^. The cooling demand^22^ for DLS was obtained from Mastrucci et al., 2019 for rural and urban households and for five world regions. For the other appliances, average annual demand values were implemented^23^.

The climate mitigation scenario assumes an advanced production energy scenario for limiting global warming well below 2 °C as implemented in the IMAGE model. It is defined by propelling high technological progress for the power sector, high electrification rates and large implementation of wind and solar sources. The scenario limits emissions to a maximum cumulative CO2 emissions and an end-of-century budget of 1150 Gt CO2 from January 2020. Also, fossil sources decline steadily and are limited to less than 20% of the primary energy mix by 2100. In IMAGE, this scenario leads to a global carbon price of 337$ per ton of carbon emitted by 2030 and to a 1.7 °C temperature increase within this century. For more information on model and scenario assumptions, see supplementary information Tables S3, S4 and S5; and Figures S5 and S6.

**Table 1:** Scenarios implemented.

Scenario name	Description		
	Universal electricity access (UA)	Climate policy	Electricity demand
BL (Baseline)	No	No	SSP2
UA-BL	Yes	No	SSP2
UA-2 C-BL	Yes	CO_2_ Emissions budget (2020–2100) < 1150 GtCO2	SSP2
UA-DLS	Yes	No	DLS (decent living standard)
UA-2 C-DLS	Yes	CO_2_ Emissions budget (2020–2100) < 1150 GtCO2	DLS

## Results

### Baseline development is not enough

**Fig. 2:**
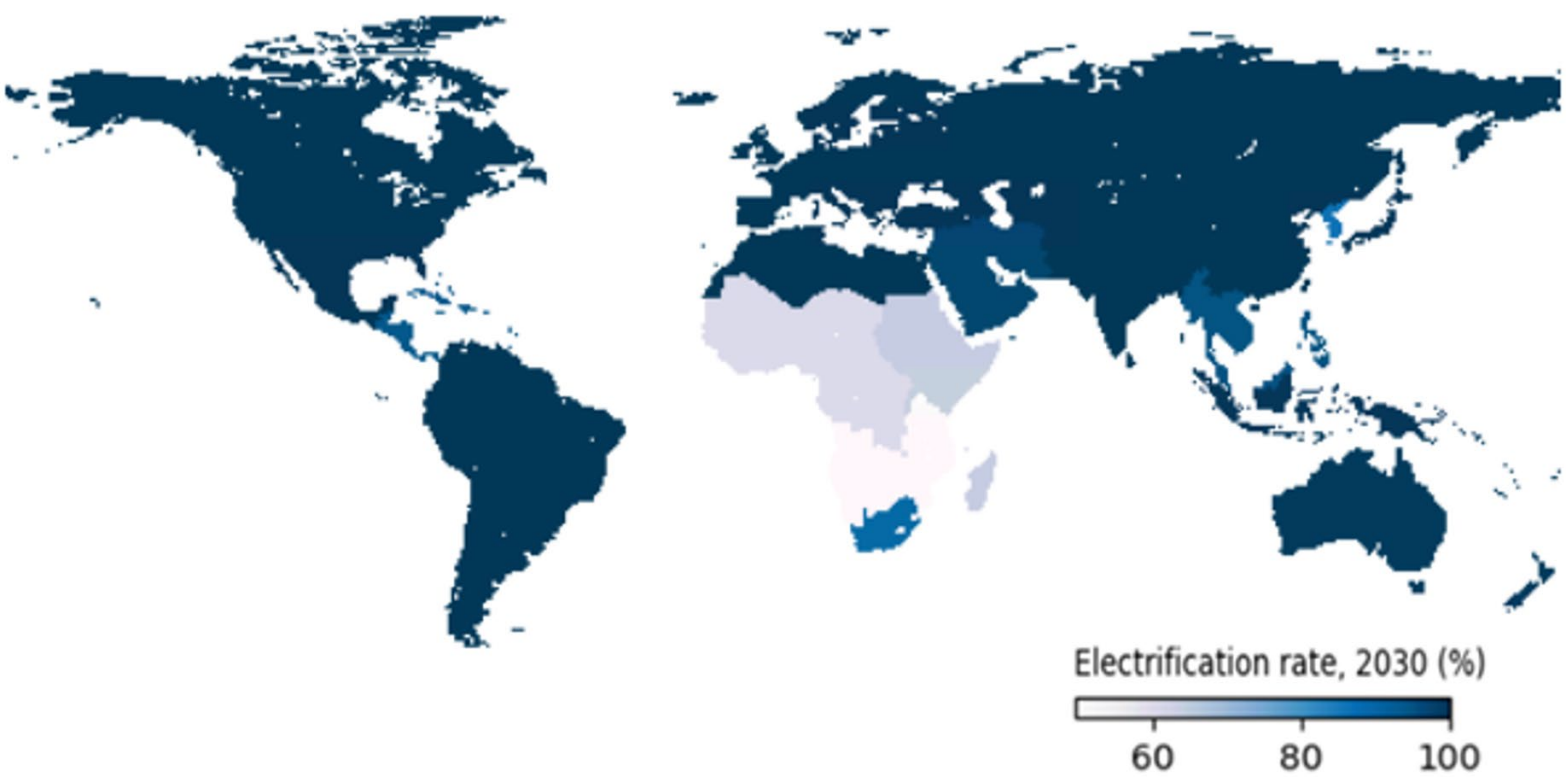
Regional percentage of the population with access to electricity in 2030 under the baseline scenario (Figure made with Plotly Python library).

In the baseline scenario, electrification rate projections are driven by income growth, population density and urbanisation trends. The modelled rates are calibrated with historical data until 2023. Under this scenario, India, North Africa, and South America are projected to achieve universal access before 2030. (Fig. 2). However, almost 600 million people are projected to still lack access by 2030, 93% of whom are located in Sub-Saharan Africa (SSA) (regional projection rates in Supplementary Information, Figure S2). Still, there has been recent progress in electrification for the Eastern Africa region, increasing the access rate projections compared to previous studies^3^. Given the low current income level and the high rural share, global access to electricity under this scenario would only be achieved by 2080, which is when the Rest of Southern Africa region achieves the goal.

In other words, SDG7.1 is not achieved. Still, the projected energy system cost is significant, as 98 billion USD annually are estimated to be invested between 2024 and 2030 in regions that currently lack full access to electricity (Table 2). This cost is mainly driven by an increase in electricity demand per household and population growth within currently electrified grid cells. Note that only around 20% of this projected cost is dedicated to SSA (Table in Supplementary Information), where the access deficit is the largest. This results from the relatively low household demand levels projected for SSA and the low progress on electrification in this scenario. The projected average access rates in this region are slightly above 60% by 2030, which mainly depends on GDP growth and urbanisation trends.

### Achieving universal access

**Fig. 3:**
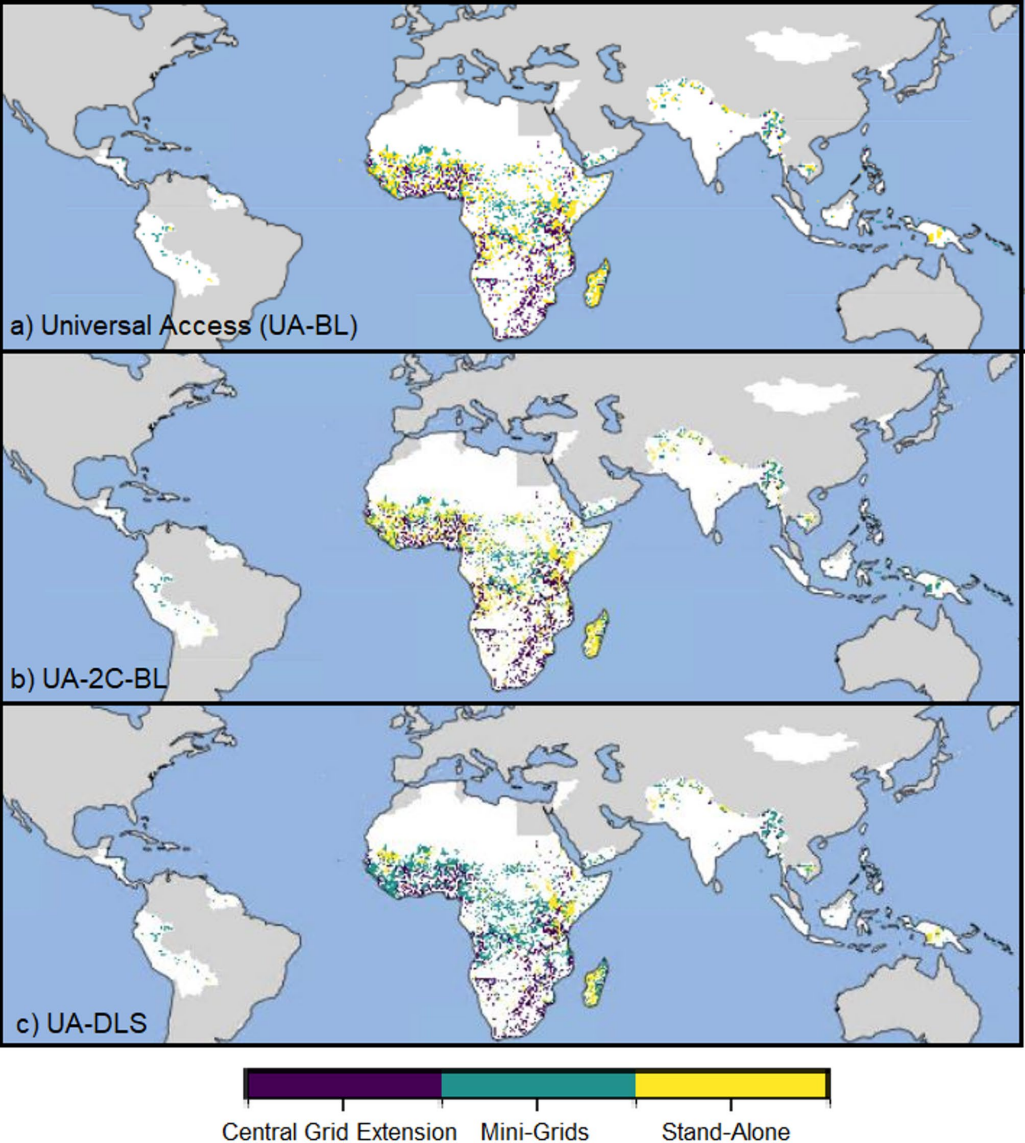
Least-cost technologies for electrification. The maps indicate cost-optimised solutions for the populated grid cells lacking access by 2023 and grouped into three categories: central-grid connection (purple dots), mini-grid connection (green dots) or stand-alone systems (yellow dots) for three scenarios. (**a**) The universal access scenario (UA-BL), (**b**) the universal access with climate policies scenario (UA-2 C-BL), (**c**) The universal access scenario targeting minimum decent living demand levels (UA-DLS). The grey areas represent countries that achieved universal access by 2023. The areas in white represent the countries without universal access in 2023. It includes populated locations that had access by 2023 and the non-populated locations. (Figure made with Plotly Python library).

**Fig. 4:**
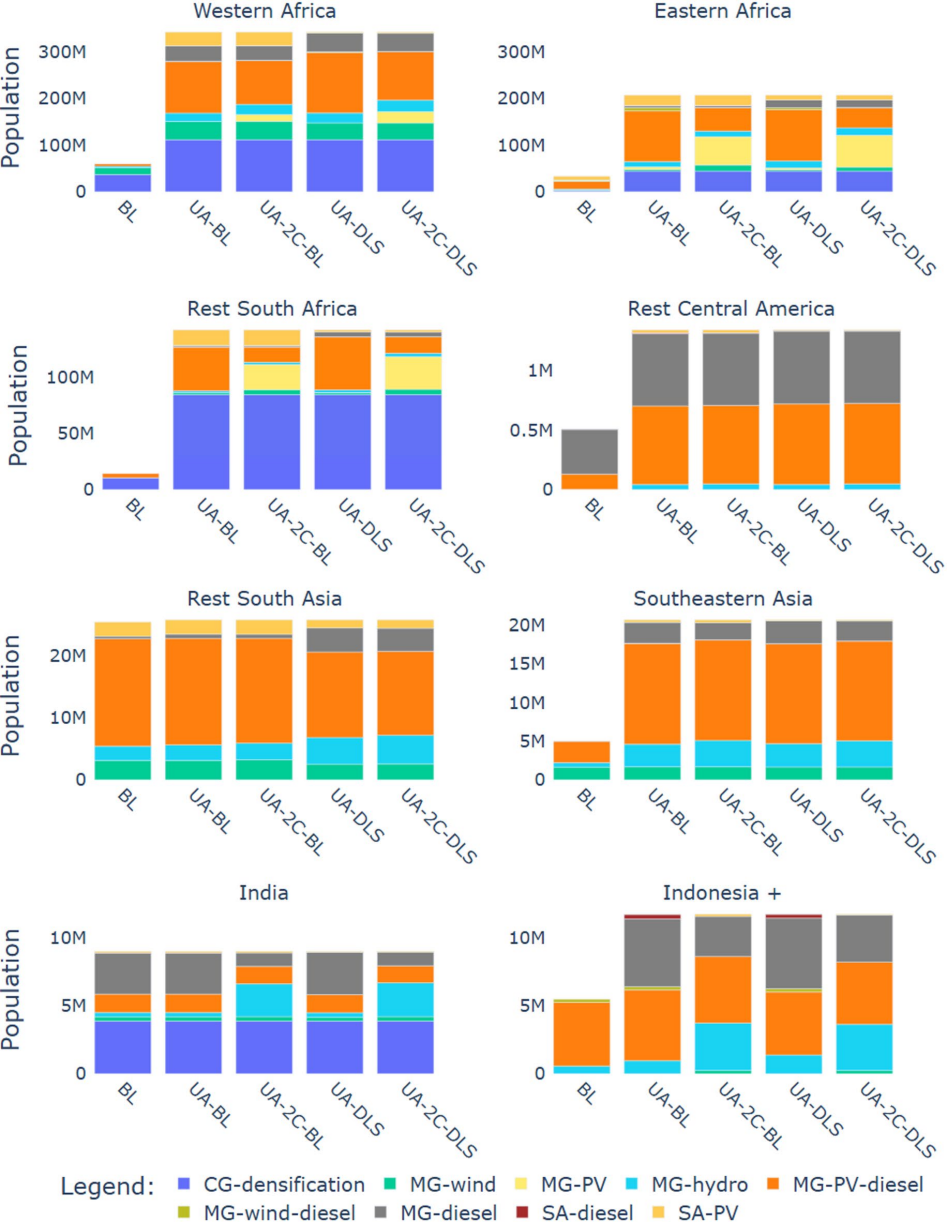
Distribution of the least-cost technologies selected over the population gaining access between 2024 and 2030 for the baseline scenario (BL) and the four universal access scenarios. Note that the vertical axes of population size vary per region.

As an alternative to the baseline, we look at four scenarios to analyse how to achieve universal access (UA) to electricity by 2030 (see Table 1 in Methods). They differ in two dimensions: (a) the level of household electricity demand achieved and (b) the presence of climate mitigation policies. In all scenarios, we allow the model to choose between different options to provide full access to electricity, either through grid extension, mini-grid solutions or stand-alone systems. The calculations consider the electricity demand density, distance to the existing grid, and local production costs. The model determines the least-cost option by comparing the LCOE of the nine options analysed for each map-grid-cell. Within 50 km of the existing central grid network, the model automatically chooses the central grid as the preferred technology to avoid revenue risk for off-grid operators and to allow the use of lower-cost middle voltage lines with a maximum length of 50 km^3,9^. The maps in Fig. 3 show the locations where central-grid extension, mini-grid connections or stand-alone systems are chosen as the least-cost options over three scenarios and intersected with the population density map, and Fig. 4 shows the optimised distributions of the nine options considered over the population gaining access between 2024 and 2030 for all four scenarios.

Results for the UA-BL scenario (without climate policies and with baseline demand) show that stand-alone (SA), mini-grid (MG) systems and central grid (CG) extensions are selected for a comparable share of locations (Fig. 3-a). Solar photovoltaics are the least-cost option for almost all SA systems selected. Only in Indonesia, some SA systems are sourced by diesel. Although SA systems were selected for many locations (typically with low population), mini-grids, especially those powered by PV-diesel combined, are the least-cost solution for the majority of people gaining access with off-grid solutions (Fig. 4). Still, SA systems remain essential to extend electricity access to low-populated areas at minimal cost. For the SSA regions and India, a larger share of the population gains access through central grid connections because there are more people lacking access within a short distance of the central grid. However, in Western and Eastern Africa, most people gain access with off-grid solutions. Comparing these results with the work of Dagnachew et al. for SSA^3^, this study, excluding the country of South Africa, projects that most people get access with off-grid solutions, while the contrary was obtained in the previous lower-resolution assessment, where central grid access dominated. Additionally, the share of mini-grids selected among the off-grid solutions is higher in the present study. These differences suggest that increasing the resolution can lead to a higher variability in the least-cost options obtained.

The climate policies implemented in the UA-2 C-BL scenario increase the share of renewables in most regions. The increase in renewables is affected by a combination of renewables enhancement policies and tax on CO_2_ emissions for the central grid connections, while the off-grid technologies were affected only by carbon taxing. Sub-Saharan Africa regions were impacted the most by the climate policies. There, the increase is dominated by solar mini-grids, while in India and Indonesia, hydropower use increases. For Rest Central America, Rest South Asia and South-Eastern Asia regions, the effect of mitigation policies on the electricity technology chosen is small. For these regions, renewable technologies are cost-competitive even in the absence of carbon taxes.

The DLS scenario (UA-DLS) shows that as a result of the increased level of demand, fewer locations (Fig. 3-c) are served by stand-alone systems (compared to the scenarios without DLS), while there is a projected increase of diesel and hybrid PV-diesel mini-grid shares. This shift from stand-alone systems to mini-grids, because of increased demand levels, is a common result in previous research for SSA^2,3^. Based on the baseline IMAGE projections of average household demand per income quintile (i.e., without implementing minimum DLS demand), 25% of the global urban population and more than 40% of the rural population cannot afford the minimum DLS demand levels. Finally, in the combined scenario with DLS and climate policies (UA-2 C-DLS scenario), the diesel demand is reduced (compared to UA-DLS) and compensated by increased use of PV mini-grids for most regions.

Globally and under all universal access scenarios, off-grid systems are selected as the least-cost solution for more than half of the population gaining access between 2024 and 2030 (almost 70% for UA scenarios), with variations of the mini-grids and stand-alone technology shares over the UA scenarios. However, the South African regions achieve universal access by mostly densifying the current grid in all scenarios because most people gaining access are located within a 50 km distance of the central grid. For many other parts of the World, mini-grids (especially sourced by PV and PV backed up by diesel) are the preferred option.

For SSA, under the UA scenarios, we project that about 75% of all people would be connected to the central grid by 2030 (note this includes people who had access by 2023). Dagnachew et al.^14^, projected that around 85% of the population would be connected to the central grid. These differences can be caused by the increased granularity of the model and the different base years (2023 and 2010).

Note that a fixed discount rate of 10% globally was implemented for all scenarios, while some regions could have higher discount rates that could influence the least-cost technology chosen. To assess the impact of the assumed discount rate on the results, we have conducted two sensitivity runs, changing the discount rate by ± 5% (figures in Supplementary Information). The system cost of regions with a larger share of central grid densification depicts higher sensitivity to changes in the discount rate. Additionally, for the variation in least-cost solutions, when reducing the discount rate, renewables, which have lower operating costs but higher capital costs, are enhanced, especially for Eastern Africa. Increasing the discount rate to 15% results in a very small increase in diesel-sourced technologies for most regions (see Supplementary Information for figures). Similar behaviour for the variations in the discount rate was seen in previous research for SSA when increasing the discount rate to higher ones^24^, as the solution with a lower capital-to-operational cost ratio was favoured. However, the central grid extension (instead of diesel off-grids) was favoured over off-grid systems for the SSA region. This difference can be related to the fact that, under this study, central grid extension is only viable within a 30 km distance for the central grid for most locations lacking access, which is lower than the 50 km distance defined as the threshold for favouring central grids (see methods). Hence, central grid selection will have less sensitivity to variations in the cost than the off-grid solutions for this study.

**Fig. 5:**
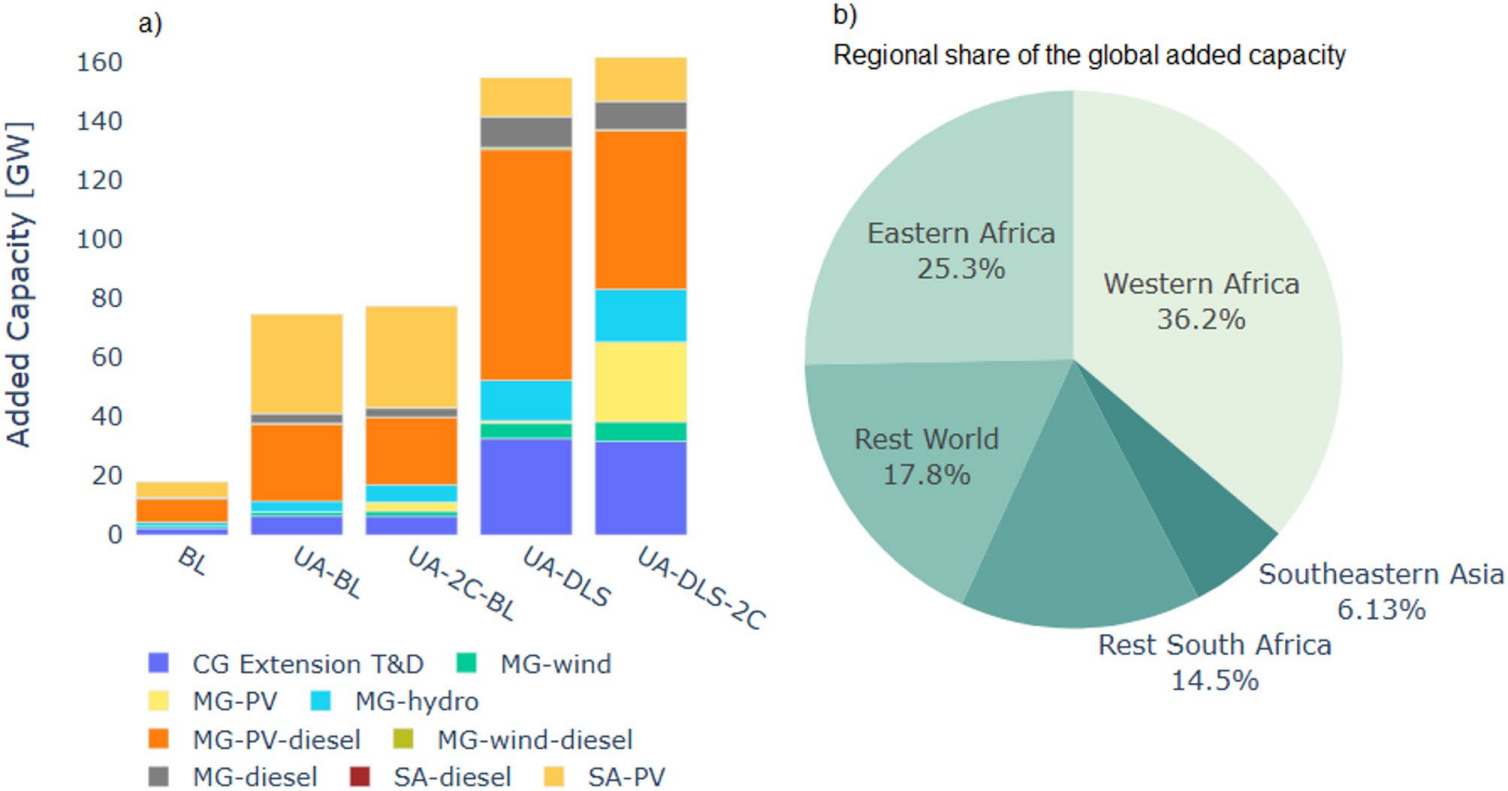
Global additional required capacity for electrification between 2024 and 2030. From left to right, plot (**a**) shows the added capacity for electrification for central grid (CG) extension and the off-grid solutions. (**b**) Shows the regional share of the global added capacity for electrification (under the DLS scenario) between 2024 and 2030.

Globally, an additional capacity of 60 GW on top of the baseline scenario (18 GW) is needed to achieve UA (Fig. 5 and Regional results in Supplementary Information, Figures S3 and S4). Moreover, this added capacity can increase to 144 GW when minimum decent living standard demand levels are considered. Off-grid systems (mainly mini-grids) provide most of this added capacity with an estimated increase of almost 40 GW of off-grid additional capacity for UA and almost 120 GW for targeting minimum DLS (Fig. 5-a).

SSA, excluding South Africa, has by far the largest share of added capacity for the electrification of new locations (Fig. 5-b). Furthermore, SSA has the largest variation across scenarios of needed capacity (more detail is presented in the supplementary material). Western Africa is the region with the largest need for capacity expansion, followed by Eastern Africa, which has the largest variation in needed capacity between the scenarios with and without the DLS consideration. For almost all regions except for South Africa, the off-grid capacity expansion increases for the DLS scenarios. For this last region, the greater demand favours the deployment of central grid extension over off-grid solutions.

For comparison purposes, we estimated the total capacity needed by SSA for 2030 (including locations with access by 2023), which ranges between 197 GW and 207 GW under the UA scenarios. Comparing this with Bazilian et al.‘s results^5^, our estimates are lower, as their estimates start at 374 GW. Though, our estimates for the DLS scenario are within range compared to the results from the IEA sustainability scenario^25^, in which they project 284GW of needed capacity by 2030 for the whole of Africa. Differences can be related to different assumptions, such as demand size projections, different storage requirements and load factors.

Table 2 summarises the results obtained under all scenarios analysed in 2030 (for the countries currently lacking universal access, i.e. white areas in Fig. 3). The additional global annual cost for achieving universal access (UA-BL) by 2030 is estimated to be 15 billion USD_2005_ on top of the baseline scenario (projected at 98 billion USD/year), and it would lead to a small (3%) increase in residential CO_2_ emissions in 2030 relative to baseline (almost 640 Mtons CO_2_). When compared to the projected global emissions for 2030, the increase is less than 0.5%. Furthermore, achieving UA to electricity and other modern energy services can have synergies with human development goals^26^, it is fundamental for ending poverty (SDG1), supporting progress on the educational level (SDG4) and reducing inequality (SDG 10). In turn, these human-related development goals could have synergies with mitigating climate change. In the climate policy scenario, electricity-related residential CO_2_ emissions can be reduced by nearly 30% with only a 5% additional cost on top of the UA scenario.

Concerning the scenarios for ensuring DLS demand for all, the cost required is almost 50% higher than the UA-BL scenario, and it would amount to an additional annual cost of a hundred billion USD on top of the baseline (BL). The cost increase is the highest in the SSA (almost doubled) because it has the lowest projected household demand levels under the baseline demand scenario. Additionally, under this scenario, the LCOE is lower for SSA (see Supplementary Information, Tables S1 and S2) due to the fixed cost for the distribution network, which depends on the length of the network needed and the number of houses per grid cell. Nevertheless, ensuring DLS can have large synergies with reducing poverty (SDG1), and this study also shows that if reaching DLS is the aspiration, different solutions might be preferred from a cost standpoint, which should be anticipated to avoid additional costs at a later stage. In the combined climate policies and DLS scenario (UA-DLS-2 C), CO_2_ emissions can be reduced by 30% with a rounded 10% additional cost compared to the UA-DLS. Implementing a carbon tax on electrification similarly impacts the cost and emission reductions under both demand-level scenarios. However, note that there is still potential for larger emissions reduction if lower electricity demand by efficiency improvement is considered^14^.

**Table 2:** Data results obtained under all scenarios analysed for 2030 globally (excluding countries that achieved universal access by 2022).

Scenario	Description	Residential electricity demand (TWh)	Share of low carbon technologies in the electricity mix (%)	CO2 emissions from residential electricity use (Mt)	Average electricity prices ($/MWh)	Annual discounted system cost required until 2030 (Billion US$_2005_/year)
BL	Baseline leading to a 93% electrification rate	1130	37	638	66	98
UA-BL	Universal Access (UA) with baseline demand	1202	35	658	75	113
UA-2 C-BL	UA with climate mitigation	1202	51	479	97	119
UA-DLS	UA and ensures decent living standard levels (DLS)	2227	33	1160	80	161
UA-2 C-DLS	UA with climate mitigation and ensures DLS	2227	50	803	102	175

**Fig. 6:**
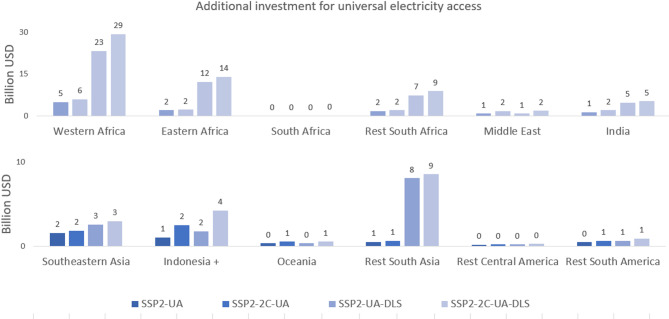
Regional comparison of the additional cost in USD 2005 (excluding baseline cost) required for achieving universal electricity access under three scenarios.

Figure 6 enables us to analyse regional differences in the additional energy system cost (on top of the baseline scenario) required for achieving universal access with and without climate mitigation and with the DLS consideration. Under the three scenarios, Western Africa is the region with the largest cost needs, followed as expected by the other regions in SSA. Rest-South Asia region and the SSA region depict the largest increase in cost under the DLS scenario. It is worth noticing that for SSA, the unitarian price of electricity increases considerably for the universal access scenarios, and therefore, the affordability aspects of electrification should also be considered in future studies.

About the mitigation scenario, most regions depict a small increase in cost. However, for Indonesia and the Middle East regions, the costs are doubled in response to the carbon tax implementation. Both regions show a switch from diesel-sourced technologies to PV stand-alones when the carbon tax is implemented (see Supplementary Information).

### Limitations and future research

The results obtained here aim to give guidance on global and regional pathways for achieving the goal. At the local level, although it can give guidance, local considerations can affect the capital cost and viability of a certain project. Furthermore, to avoid loss of capital for off-grid systems in the long term because of central grid extension, off-grid systems should be built considering future central grid connections. The investment needs for achieving universal access largely depend on the system size. For the decent living standard scenario, a good exercise would be to fix the electricity demand to the DLS regional values (instead of using the DLS values as a minimum demand). This would reduce the cost of achieving the goal while ensuring that all people gain power connections to a sufficient capacity level.

For this assessment, a fixed discount rate is implemented, enabling analysis of the deployment of central-grid versus off-grid solutions regardless of possible differences in discount rate variations across regions and between private and public investors. Based on the sensitivity test conducted, when reducing the discount rate, renewables with lower operation costs are enhanced for some regions, while the opposite occurs with a higher discount rate (See supplementary information, Figure S7 and Table S6). Furthermore, some variables used in the analysis carry uncertainties that can be explored further through sensitivity assessments or a probabilistic analysis. These include uncertainties in the potential RE maps implemented, projected electricity demand levels, and the consumption density threshold applied to mini-grids and stand-alone systems. For this last one, a higher threshold implies lower numbers of mini-grids selected^27^. Finally, the affordability aspects of electricity use and the governance required are outside the scope of this study and could influence the progress and the selection of solutions for achieving the goal. For the affordability aspects, a future study can include the financing instruments and the commercial uses of electricity.

## Conclusions and discussion

This study analyses the least-cost pathways for achieving universal electricity access globally by 2030, starting policy implementation in 2024, and in line with the current SDG goal. Clearly, by now achieving this goal in the remaining five years is practically impossible, especially considering the time required to set up the necessary investment projects. In fact, ensuring universal energy access is by itself a formidable challenge, despite previous success in specific countries. For instance, lack of infrastructure, weak regulatory environments, political instability and limited technical capacity in the relevant countries (mainly for Sub-Saharan Africa) could seriously hinder progress^28,29^. It is also known that implementation delays played a role in previous electrification efforts^30–32^. In other words, in addition to the technical and financial considerations discussed in this paper, a whole set of other conditions need to be assured to make universal access, as promised in the SDGs, feasible. Hence, the scenarios proposed here should be viewed as a demonstration of the scale and speed of the technological and economic effort required for achieving the original goal. By recognising these challenges, our study aims to highlight the urgency and the substantial commitment necessary from all stakeholders involved. This can also be used as input into reformulations of the SDG goals in the coming years.

The scenarios look into baseline development, implementation of decent living standards and the synergies with climate change mitigation. We look at nine electrification technologies grouped into grid extension, six mini-grid solutions and two stand-alone systems. The model integrates regional energy projections and higher-resolution grid data. This improves the assessment of the regional least-cost technology mix for electrification based on more granularity of the local energy resources and population needs. The results help to identify the best strategies for electrification.

### The study shows that in the absence of additional electrification policies, SDG7.1 is not achieved

In this baseline scenario, almost 600 million people are projected to still lack access by 2030, 93% of whom are located in Sub-Saharan Africa (SSA). In fact, universal access may only be achieved by 2080 under baseline assumptions (based on past trends).

### Targeting UA leads to at least 50GW of off-grid additional capacity needed, most of which is required for SSA

 Off-grid systems, i.e. mini-grids and solar home systems, are the least-cost solution for most people gaining access between 2024 and 2030. Under baseline consumption levels, about 20% of the global urban and more than 40% of the global rural population would get access to levels below decent living standards (based on the projections of average household demand per income quintiles). If the ambition is targeting universal access and achieving decent living standards, the additional off-grid capacity would almost double, with a preference for mini-grids over stand-alone systems. The strongest increase in that scenario would be in Eastern Africa because of its very low projected household demand under baseline conditions.

### The combination with climate policies leads to a strong increase in renewables deployment

 The higher share of renewable-based systems (both for central grid and off-grid connections) would result in a significant reduction in carbon emissions (by 30%) with a small increase in cost compared to the default UA scenario. The mitigation policies analysed here focus on reducing emissions from fossil fuel switching and do not consider reducing household demand by efficiency improvement. Therefore, there is potential for larger emissions reduction. Also, it should be considered that although the increase in cost is low, it can lead to an increase in electricity prices. Hence, there is a need for complementary policies to protect people with low incomes from higher electricity prices.

### The additional global annual cost for achieving universal access by 2030 on top of the baseline scenario (BL) is estimated to be between 15 billion (only universal access) and almost 80 billion USD for the combined policy scenario that achieves UA with climate change mitigation and the DLS consideration (UA-DLS-2 C)

 The most important factor determining costs is the total system size: if policies aim to achieve decent living standards, this requires a substantial increase in capacity and, thus, costs. Therefore, if governments prioritise universal access at a lower demand level due to capacity limitations, systems should be built to accommodate capacity expansion and enable system integration. The impact of universal access policies and even the addition of climate policy leads to smaller increases in cost levels at the global scale. Finally, although the total cost for the DLS scenario is larger, the LCOE is lower in the SSA region, which can help people afford the DLS demand levels.

## Supplementary Information

Below is the link to the electronic supplementary material.

Supplementary Material 1

## Data Availability

The datasets generated during the current study are available in the Mendeley Data repository, doi: 10.17632/5y37jthhrr.1 (https://data.mendeley.com/datasets/5y37jthhrr/1).

## Code availability

The code is available at https://github.com/victhaliaz/SDG7_global_electrification/.
