# Supplementary material for: High-resolution global pathways to achieve 100% electricity access in 2030
Source: Sci Rep. 2025 Nov 17;15:40096. doi: 10.1038/s41598-025-23857-4 (PMC12623783; doi:10.1038/s41598-025-23857-4)
Supplement: Supplementary file 1 — Supplementary Material 1 [file 41598_2025_23857_MOESM1_ESM.docx]

# Global Pathways for Universal Electricity Access in 2030

# Supplementary Information

## Regional classification


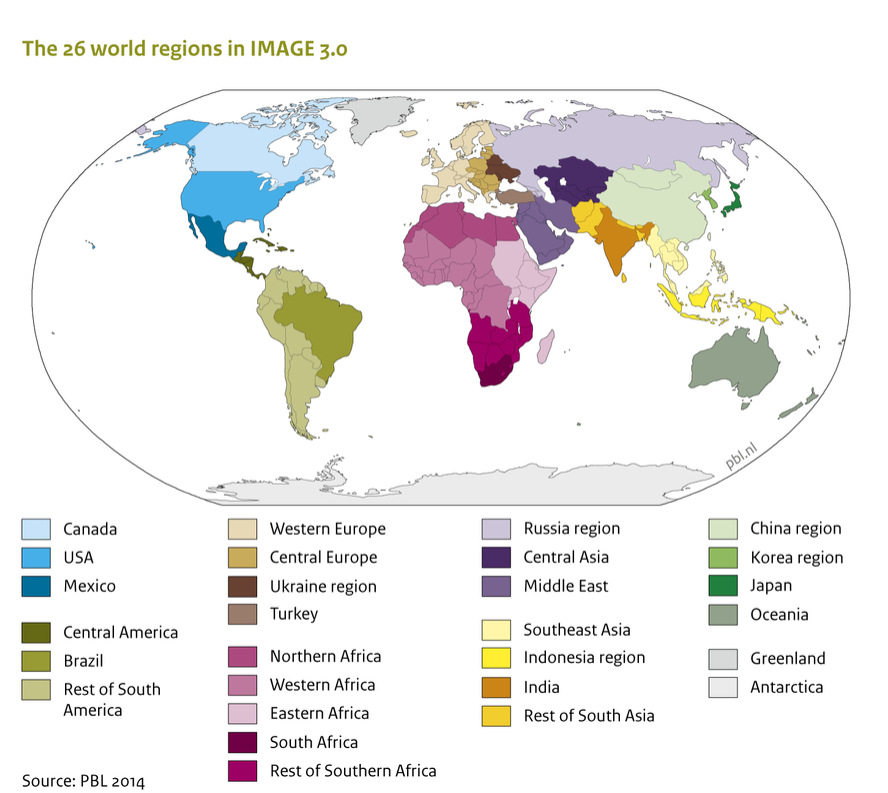


Figure S1 The IMAGE framework region classification.

## Further results


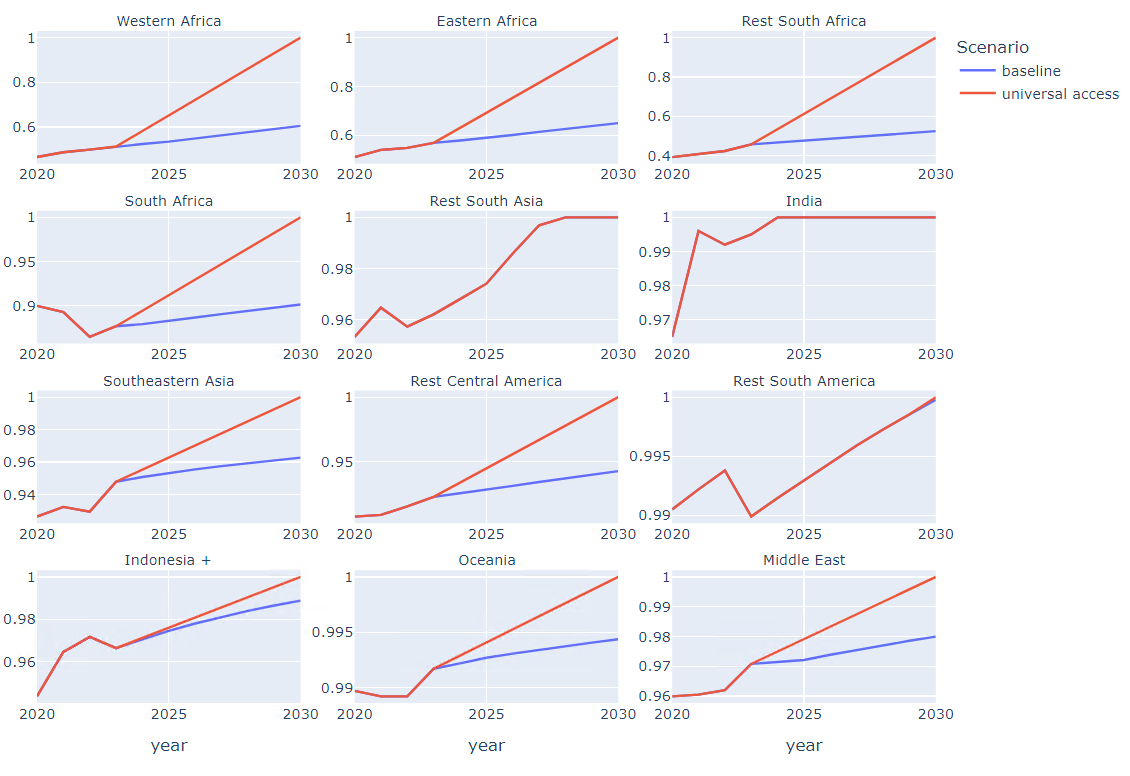


Figure S2 Electrification rate projections under the SSP2 baseline scenario (blue) and for achieving universal access by 2030 (red)


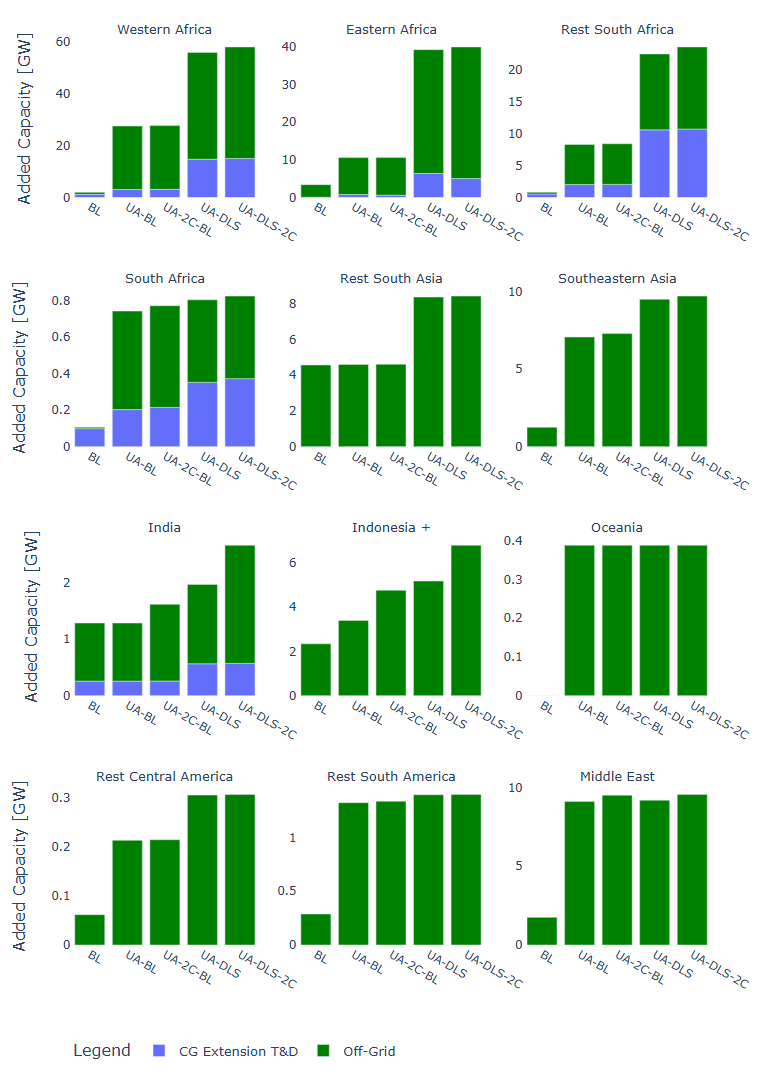


Figure S3 Estimated Increased in installed capacity required for electrification under the different scenarios between 2024 to 2030 for the new central grid (blue) and off-grid connections (green).


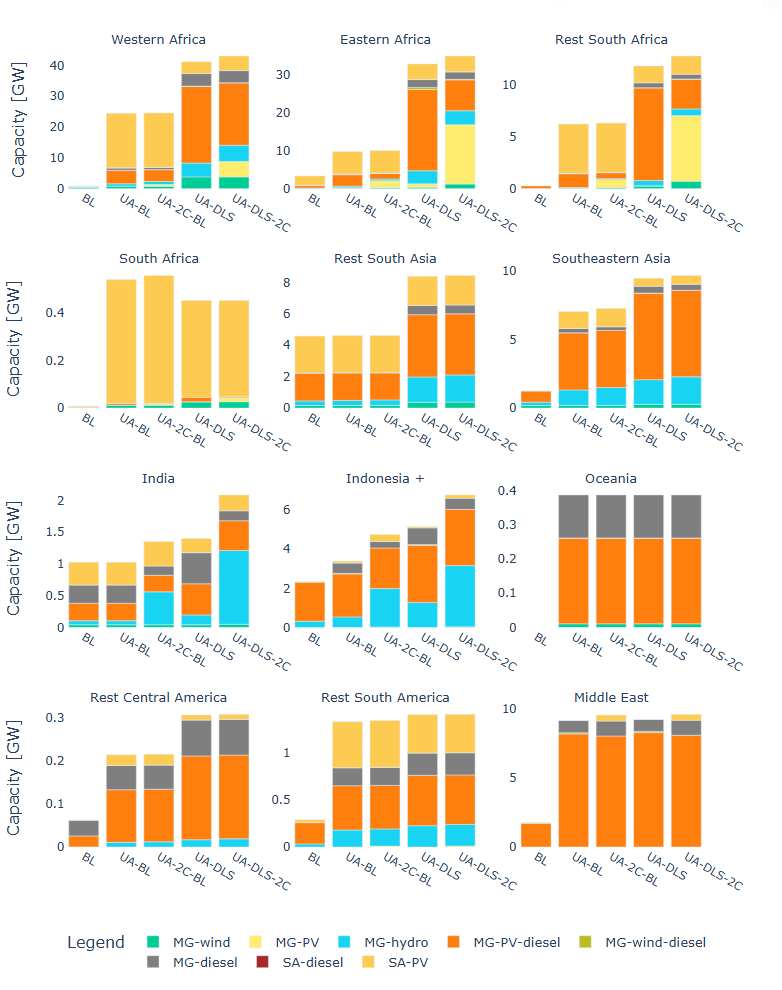


Figure S4 Estimated Increased installed capacity required for electrification under the different scenarios between 2024 to 2030 for the new off-grid installed technologies.

Table S1 Data results obtained under all scenarios analysed for 2030 for the Sub Sahara Africa region

| Scenario | Description | Residential electricity demand (TWh) | Share of low carbon technologies in the electricity mix (%) | CO2 emissions from residential electricity use (Mt) | Average electricity prices ($/MWh) | Annual discounted system cost required until 2030 (Billion US$_2005_/year) |
| --- | --- | --- | --- | --- | --- | --- |
| BL | Baseline leading to a 61% electrification rate | 103 | 46 | 45 | 82 | 24 |
| UA­­ | Universal Access (UA) with baseline demand | 144 | 41 | 54 | 133 | 33 |
| UA-2C | UA with climate mitigation | 144 | 57 | 33 | 159 | 34 |
| UA-DLS | UA and ensures decent living standard levels (DLS) | 569 | 36 | 169 | 115 | 59 |
| UA-DLS-2C | UA with climate mitigation and ensures DLS | 569 | 56 | 98 | 139 | 76 |

Table S2 The annual regional system cost for electrification until 2030 for the scenarios analysed in USD 2005.

| ***region*** | **SSP2-BL** | **SSP2-UA** | **SSP2-2C-UA** | **SSP2-UA-DLS** | **SSP2-2C-UA-DLS** |
| --- | --- | --- | --- | --- | --- |
| Western Africa | 10.55613 | 15.55801 | 16.55881 | 33.85776 | 39.90202 |
| Eastern Africa | 8.012048 | 10.14686 | 10.29088 | 20.31401 | 22.02738 |
| South Africa | 1.948164 | 2.039966 | 2.085884 | 2.106976 | 2.187416 |
| Rest South Africa | 3.135744 | 5.006841 | 5.229954 | 10.55597 | 12.10384 |
| Middle East | 9.024641 | 9.975938 | 10.87779 | 9.972686 | 10.89983 |
| India | 34.68075 | 36.05626 | 36.7839 | 39.54889 | 39.99212 |
| Southeastern Asia | 6.314933 | 7.884949 | 8.163594 | 8.868934 | 9.285477 |
| Indonesia + | 7.389977 | 8.423198 | 9.875862 | 9.168321 | 11.62117 |
| Rest South Asia | 11.52563 | 12.03591 | 12.13865 | 19.61648 | 20.09105 |
| Rest Central America | 2.995719 | 3.154506 | 3.202873 | 3.195919 | 3.261349 |
| Rest South America | 2.183725 | 2.646896 | 2.823947 | 2.82015 | 3.06421 |

## General assumptions

The transmission and distribution capacity for central grid connections or mini-grids were calculated using a threshold on the average household demand per region. Regions with low household demand (lower than 700kWh/year) are assumed to use electricity for four hours daily. While for regions with average household demand exceeding this threshold, the peak demand was implemented using a residential load curve obtained from Zapata et al., [1]

Other techno-economic assumptions are listed below, and the remaining assumptions were left as described in Dagnechew et al., [2]

Table S3 General Assumptions

| **Description** | **Value** |
| --- | --- |
| Discount rate for net present value [Standard for IMAGE scenarios] | 10% |
| CO2 emissions of diesel generators [3] | 2.68 kgCO_2_/liter |
| Diesel consumption in generators [4] | 0.266 liters/kWh |
| Diesel generator stand -alone life-time [5] | 10 years |
| Diesel cost: world-bank country data for retail price [6] and trended with IMAGE scenario results. |  |
| Technical lifetime of solar wind and diesel mini-grids [5] | 25 years |
| Storage kwh per system kilowatt for solar systems [2] | 4 hours |
| Storage kwh per system kilowatt for wind systems [2] | 2 hours |
| Battery lifetime [7] | 10 years |

Figure S5 Carbon tax implemented for the mitigation scenarios for selected regions from the IMAGE model as given from the NAVIGATE project [8].


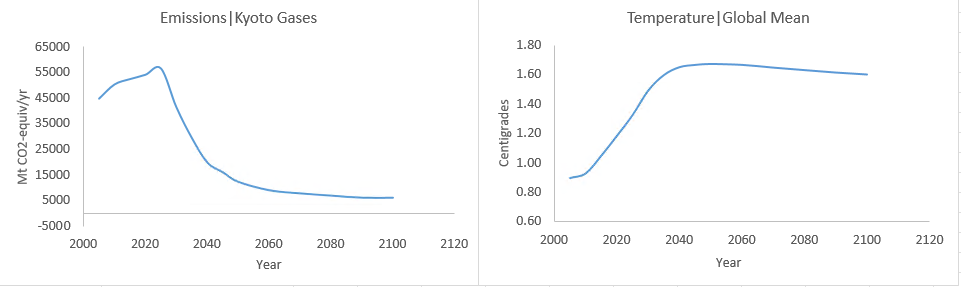


Figure S6 Annual global green house gases emissions and temperature projections under the mitigation scenarios from the IMAGE model as given from the NAVIGATE project. The data available has a five-year time-step, hence the COVID-19 effect is not visible with this resolution [8].

### Household electricity demand data

Table S4 Annual household electricity demand projections for 2030 per income quintile from IMAGE-TIMER

|  | **IMAGE-TIMER projection for 2030 [kWh]** | | | | | | | | | |
| --- | --- | --- | --- | --- | --- | --- | --- | --- | --- | --- |
| **Regions** | **Urban Q1** | **Urban Q2** | **Urban Q3** | **Urban Q4** | **Urban Q5** | **Rural Q1** | **Rural Q2** | **Rural Q3** | **Rural Q4** | **Rural Q5** |
| Canada | 9113.4 | 10721.3 | 11862.3 | 12849.4 | 14242.2 | 10126.5 | 11503.0 | 12568.0 | 13673.5 | 15086.3 |
| USA | 4909.9 | 7710.5 | 10921.2 | 13857.2 | 18966.4 | 5735.3 | 5983.7 | 9658.0 | 12931.2 | 18284.5 |
| Mexico | 1156.7 | 1534.8 | 1927.2 | 2338.1 | 2917.1 | 1147.2 | 1330.3 | 1571.0 | 2454.8 | 3048.5 |
| Rest Central America | 967.9 | 1205.8 | 1446.6 | 1944.3 | 3010.8 | 539.5 | 677.2 | 815.2 | 1024.4 | 1920.2 |
| Brazil | 1176.3 | 1441.8 | 1663.2 | 1965.4 | 3263.5 | 1046.5 | 1255.4 | 1410.5 | 1595.6 | 2193.5 |
| Rest South America | 666.0 | 942.7 | 1157.0 | 1451.6 | 2307.2 | 586.9 | 804.1 | 1012.6 | 1381.3 | 2157.1 |
| Northern Africa | 1819.1 | 2298.9 | 2375.0 | 2666.9 | 3480.3 | 1300.9 | 1615.3 | 1991.4 | 2331.2 | 2815.6 |
| Western Africa | 290.4 | 392.1 | 494.0 | 625.0 | 999.6 | 110.2 | 204.5 | 297.6 | 399.5 | 569.9 |
| Eastern Africa | 216.2 | 299.6 | 385.3 | 510.3 | 962.3 | 100.3 | 161.9 | 227.3 | 303.5 | 454.5 |
| Southern Africa | 974.0 | 1630.3 | 1916.6 | 2350.2 | 3225.9 | 536.1 | 715.0 | 894.9 | 1096.0 | 1486.3 |
| Western Europe | 3487.2 | 3960.3 | 4295.0 | 4605.2 | 4999.5 | 3359.4 | 3738.6 | 4054.9 | 4362.7 | 4773.0 |
| Central Europe | 2620.7 | 3066.4 | 3376.1 | 3640.0 | 4003.8 | 2119.6 | 2361.6 | 2626.4 | 2843.6 | 3187.1 |
| Turkey | 1746.2 | 2095.5 | 2422.7 | 2725.2 | 3238.5 | 2417.2 | 3277.5 | 4126.2 | 4995.2 | 5362.2 |
| Ukraine + | 1333.8 | 1550.0 | 1768.8 | 2055.6 | 2532.0 | 1581.6 | 1982.3 | 2270.9 | 2572.3 | 3083.0 |
| Asia-Stan | 755.8 | 898.3 | 1017.3 | 1168.9 | 1630.4 | 729.4 | 863.5 | 975.5 | 1095.7 | 1399.5 |
| Russia + | 1549.6 | 1799.6 | 2039.0 | 2268.0 | 2637.8 | 1384.0 | 1549.1 | 1736.2 | 1984.6 | 2365.5 |
| Middle East | 2049.9 | 3689.7 | 4432.3 | 4863.4 | 5458.5 | 1898.9 | 3187.1 | 4158.8 | 4646.7 | 5039.3 |
| India + | 856.8 | 1160.9 | 1578.1 | 2208.5 | 4036.0 | 675.3 | 812.9 | 952.4 | 1149.3 | 1756.5 |
| Korea | 2296.9 | 3037.3 | 3210.1 | 3581.0 | 4053.7 | 1129.1 | 1414.2 | 1809.5 | 2842.2 | 3292.9 |
| China + | 2477.4 | 3014.6 | 3417.2 | 3787.2 | 4309.4 | 1266.2 | 1635.9 | 1894.7 | 2127.5 | 2670.4 |
| Southeastern Asia | 1052.7 | 1294.4 | 1518.2 | 1823.3 | 2503.5 | 907.5 | 1157.5 | 1381.4 | 1612.0 | 2036.5 |
| Indonesia + | 902.6 | 1097.8 | 1288.7 | 1515.4 | 2107.8 | 809.1 | 990.7 | 1151.0 | 1315.9 | 1542.1 |
| Japan | 3845.5 | 4977.7 | 5647.7 | 6243.0 | 7142.8 | 4236.6 | 5240.0 | 6021.4 | 6727.8 | 7723.0 |
| Oceania | 3714.3 | 4313.6 | 4714.7 | 5076.2 | 5542.6 | 3411.4 | 3965.1 | 4420.1 | 4875.2 | 5454.6 |
| Rest S.Asia | 665.4 | 833.1 | 982.6 | 1124.8 | 1288.1 | 530.5 | 657.9 | 798.7 | 973.6 | 1212.7 |
| Rest S.Africa | 387.3 | 513.4 | 648.1 | 829.5 | 1304.4 | 91.5 | 199.8 | 298.5 | 390.0 | 498.7 |

Table S5 Minimum annual household electricity demand estimation for a decent living.

|  | **DLS [kWh]** | |
| --- | --- | --- |
| **Regions** | **Urban** | **Rural** |
| Canada | 1093.2 | 1181.1 |
| USA | 1174.3 | 1313.8 |
| Mexico | 1155.6 | 1521.9 |
| Rest Central America | 1156.2 | 1525.8 |
| Brazil | 1165.4 | 1548.7 |
| Rest South America | 1130.2 | 1449.8 |
| Northern Africa | 1373.8 | 1842.1 |
| Western Africa | 1664.6 | 2461.7 |
| Eastern Africa | 1465.7 | 2046.9 |
| Southern Africa | 1359.6 | 1810.1 |
| Western Europe | 1113.3 | 1214.3 |
| Central Europe | 1133.9 | 1249.0 |
| Turkey | 1152.4 | 1279.1 |
| Ukraine + | 1125.1 | 1234.3 |
| Asia-Stan | 1079.5 | 1160.8 |
| Russia + | 1160.7 | 1292.4 |
| Middle East | 1337.8 | 1763.2 |
| India + | 1761.2 | 2503.7 |
| Korea | 1143.4 | 1263.8 |
| China + | 1087.1 | 1172.8 |
| Southeastern Asia | 1608.7 | 2206.7 |
| Indonesia + | 1444.8 | 1937.3 |
| Japan | 1398.6 | 1839.8 |
| Oceania | 1336.6 | 1713.4 |
| Rest S.Asia | 1846.0 | 2666.7 |
| Rest S.Africa | 1578.5 | 2282.6 |

## Sensitivity runs

We have conducted two runs for analysing the sensitivity of the results to a change on the global discount rate on the universal access scenario (SSP2-UA). We have increased and reduced the discount rate by 5% compared to the default global value of 10%.

Overall, when reducing the discount rate, renewables, which have lower operating costs but larger capital costs, are enhanced, especially for Eastern Africa. Increasing the discount rate to 15% results in a very small increase in diesel-sourced technologies or hydropower for the Indonesia case.


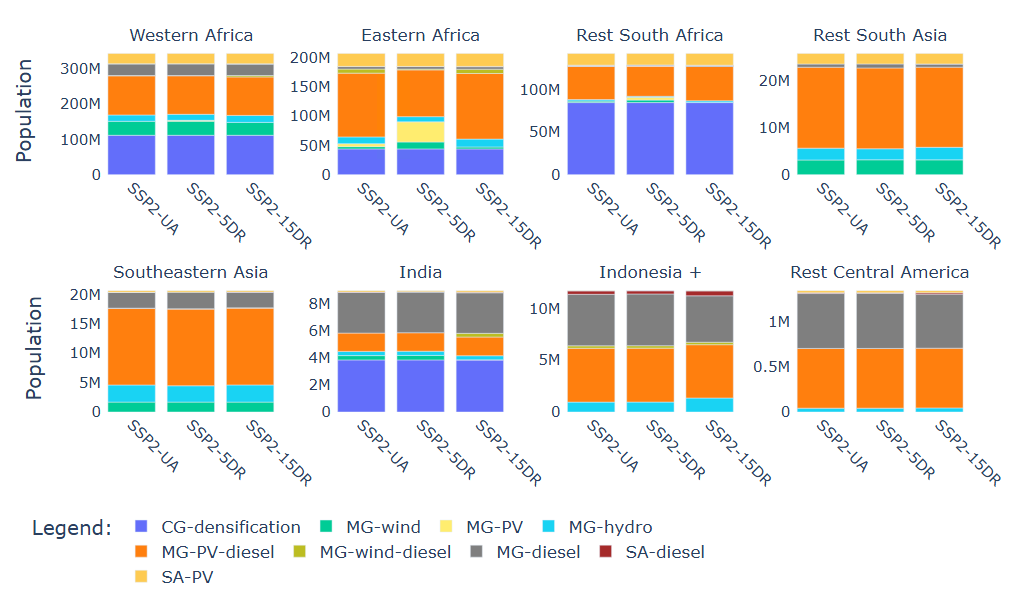


Figure S7: Distribution of the least-cost technologies selected over the population gaining access between 2024 and 2030 for the universal access scenario (SSP2-UA) with a 10% discount rate and the variation of this to a 5% (SSP2-5DR) and 15% (SSP2-15DR) discount rate. Note that the vertical axes of population size vary per region.

Table S6 Sensitivity assessment of the annual regional system cost for electrification until 2030 in USD 2005.

| region | SSP2-UA | SSP2-5DR | variation (%) | SSP2-15DR | variation (%) |
| --- | --- | --- | --- | --- | --- |
| Rest Central America | 3.154506 | 3.131022 | -0.744474225 | 3.180748 | 0.831878 |
| Rest South America | 2.646896 | 2.608655 | -1.444741164 | 2.688994 | 1.590487 |
| Western Africa | 15.55801 | 14.49802 | -6.813139631 | 16.73628 | 7.573347 |
| Eastern Africa | 10.14686 | 9.603613 | -5.353830111 | 10.74185 | 5.863748 |
| South Africa | 2.039966 | 2.006834 | -1.624142935 | 2.077499 | 1.839895 |
| Middle East | 9.975938 | 9.723304 | -2.532428225 | 10.24056 | 2.652578 |
| India | 36.05626 | 29.74088 | -17.51532917 | 36.87805 | 2.279207 |
| Southeastern Asia | 7.884949 | 7.689699 | -2.476244617 | 8.098364 | 2.706614 |
| Indonesia + | 8.423198 | 8.234096 | -2.245007039 | 8.632061 | 2.479621 |
| Oceania | 0.448281 | 0.431305 | -3.786856676 | 0.467068 | 4.190855 |
| Rest South Asia | 12.03591 | 11.85594 | -1.495278946 | 12.2363 | 1.664989 |
| Rest South Africa | 5.006841 | 4.476565 | -10.59102216 | 5.60168 | 11.88052 |

## References

[1] Zapata Castillo V, Boer HS de, Muñoz RM, Gernaat DEHJ, Benders R, van Vuuren D. Future global electricity demand load curves. Energy 2022;258:124741. https://doi.org/10.1016/J.ENERGY.2022.124741.

[2] Dagnachew AG, Lucas PL, Hof AF, Gernaat DEHJ, de Boer HS, van Vuuren DP. The role of decentralized systems in providing universal electricity access in Sub-Saharan Africa – A model-based approach. Energy 2017;139:184–95. https://doi.org/10.1016/J.ENERGY.2017.07.144.

[3] GHG Protocol. Emission Factors Worksheet 2022. https://doi.org/https://ghgprotocol.org/calculation-tools-and-guidance#cross_sector_tools_id.

[4] S. Pelland, D. Turcotte GC and AS. Nemiah Valley Photovoltaic-Diesel Mini-Grid: System Performance and Fuel Saving Based on One Year of Monitored Data. IEEE Trans Sustain Energy 2012;3:167–75. https://doi.org/10.1109/TSTE.2011.2170444.

[5] Taliotis C, Shivakumar A, Ramos E, Howells M, Mentis D, Sridharan V, et al. An indicative analysis of investment opportunities in the African electricity supply sector — Using TEMBA (The Electricity Model Base for Africa). Energy Sustain Dev 2016;31:50–66. https://doi.org/10.1016/J.ESD.2015.12.001.

[6] World Bank. Pump price for diesel fuel n.d. https://doi.org/https://data.worldbank.org/indicator/EP.PMP.DESL.CD.

[7] Peña Balderrama JG, Balderrama Subieta S, Lombardi F, Stevanato N, Sahlberg A, Howells M, et al. Incorporating high-resolution demand and techno-economic optimization to evaluate micro-grids into the Open Source Spatial Electrification Tool (OnSSET). Energy Sustain Dev 2020;56:98–118. https://doi.org/10.1016/J.ESD.2020.02.009.

[8] NAVIGATE -h2020. (work prepared for publication) n.d. https://data.ece.iiasa.ac.at/navigate.
